# Comprehensive analysis of the cancer driver genes in breast cancer demonstrates their roles in cancer prognosis and tumor microenvironment

**DOI:** 10.1186/s12957-021-02387-z

**Published:** 2021-09-10

**Authors:** Xiao-wei Du, Gao Li, Juan Liu, Chun-yan Zhang, Qiong Liu, Hao Wang, Ting-song Chen

**Affiliations:** 1 Jinzhou Medical University, Jinzhou, China; 2 Department of Oncology, Seventh People’s Hospital of Shanghai University of Traditional Chinese Medicine, Shanghai, 200000 China; 3 Department of Pharmacy, Seventh People’s Hospital of Shanghai University of Traditional Chinese Medicine, Shanghai, China; 4 Department of Central Laboratory, Seventh People’s Hospital of Shanghai University of Traditional Chinese Medicine, Shanghai, China; 5 Office of Academic Research, Seventh People’s Hospital of Shanghai University of Traditional Chinese Medicine, Shanghai, China

**Keywords:** Cancer driver genes, Prognostic signature, Breast cancer, Tumor microenvironment, TCGA

## Abstract

**Background:**

Breast cancer is the most common malignancy in women. Cancer driver gene-mediated alterations in the tumor microenvironment are critical factors affecting the biological behavior of breast cancer. The purpose of this study was to identify the expression characteristics and prognostic value of cancer driver genes in breast cancer.

**Methods:**

The Cancer Genome Atlas (TCGA) and Gene Expression Omnibus (GEO) datasets are used as the training and test sets. Classified according to cancer and paracancerous tissues, we identified differentially expressed cancer driver genes. We further screened prognosis-associated genes, and candidate genes were submitted for the construction of a risk signature. Functional enrichment analysis and transcriptional regulatory networks were performed to search for possible mechanisms by which cancer driver genes affect breast cancer prognosis.

**Results:**

We identified more than 200 differentially expressed driver genes and 27 prognosis-related genes. High-risk group patients had a lower survival rate compared to the low-risk group (*P*<0.05), and risk signature showed high specificity and sensitivity in predicting the patient prognosis (AUC 0.790). Multivariate regression analysis suggested that risk scores can independently predict patient prognosis. Further, we found differences in PD-1 expression, immune score, and stromal score among different risk groups.

**Conclusion:**

Our study confirms the critical prognosis role of cancer driver genes in breast cancer. The cancer driver gene risk signature may provide a novel biomarker for clinical treatment strategy and survival prediction of breast cancer.

## Background

In the past few years, the incidence of breast cancer has been on the rise. Among all malignant tumors in women, breast cancer ranks first with an incidence rate of 30% [1]. Women with breast cancer have the second-highest mortality rate, and the prognosis of patients with different molecular types varies significantly [2–4]. For example, the choice of drugs varies among patients with different hormone receptor status, and patients with triple-negative breast cancer usually have a poorer prognosis [5–7]. Besides, traditional tumor staging sometimes cannot objectively represent the tumor status of patients. Patients with the same tumor stage sometimes have significant survival differences [8]. The heterogeneity of breast cancer poses a challenge for clinical treatment strategy choice and prognosis prediction, while studies of different molecular typing are expected to elucidate the differences in heterogeneity and better guide clinical treatment of breast cancer.

The journal of *Nature Reviews Cancer* recently reported on the important role played by cancer driver genes in the progression of tumor malignancy [9]. 568 cancer driver genes were identified through a large-scale transcriptome analysis, which may mediate complex molecular regulatory networks and changes in the tumor microenvironment. Aberrantly expressed cancer driver genes may result in multiple processes such as uncontrolled tumor cell proliferation, invasion, recurrence, and drug resistance [10, 11]. Zhang’s study reported the frequency of mutations in the whole genes of breast cancer [12]. The most frequently mutated genes were TP53 (45%), followed by PIK3CA (44%), GATA3 (18%), and MAP3K1 (10%), all of which also were identified as cancer driver genes. Kruse’s study [13] identified metastasis driver genes in breast cancer by massive parallel sequencing, and the cancer driver genes DCC and CREBBP were also identified as key metastatic genes.

Tumorigenesis is often associated with alterations in the stromal environment and immune status, mainly manifested as changes in the tumor microenvironment (TME) [14, 15]. TME plays a key role in several steps of tumor development, including local drug resistance, immune escape, recurrence, and distant metastasis [16, 17]. Tumor cell exposure to TME also contributes to shaping the tissue-specificity of driver genes [18]. Antibodies or inhibitors that target driver gene-mediated signaling pathways may effectively inhibit tumor growth and prolong patient survival [19]. KRAS mutations may affect the TME and patient response to immunotherapy [20]. Cancer driver genes and TME determine the type of liver cancer and can be considered as predictors of patient survival outcomes [21, 22]. All these studies provide a reference for clinical decisions. However, there are fewer studies on cancer driver genes affecting the TME and prognosis of breast cancer.

In this study, we systematically analyzed the expression characteristics of 568 cancer driver genes in breast cancer. We screened for risk driver molecules that affect breast cancer prognosis, and the relationship between risk groups with the tumor microenvironment. Our study is expected to find novel molecular subtypes of breast cancer for better guiding clinical treatment strategies.

## Materials and methods

### Data sources

Breast cancer transcriptome data and corresponding clinical data were downloaded from The Cancer Genome Atlas (TCGA) (https://portal.gdc.cancer.gov/) database. As a training set, TCGA transcriptome data contained a total of 112 cases of paracancerous tissues and 1096 cases of cancerous tissues, and the clinical information of patients is shown in Table 1.

**Table 1:** Clinical features of BRCA patients (*n* = 1096) from TCGA database

Variables	*N*	%
Total	1096	100.0%
Age, years		
<60	582	53.1%
≥60	514	46.9%
Stage		
Stage i	183	16.7%
Stage ii	621	56.7%
Stage iii	248	22.6%
Stage iv	20	1.8%
T classification		
T1	281	25.6%
T2	635	57.9%
T3	138	12.6%
T4	39	3.6%
ER status		
Negative	796	72.6%
Positive	233	21.3%
PR status		
Negative	688	62.8%
Positive	338	30.8%

The Gene Expression Omnibus (GEO) (https://www.ncbi.nlm.nih.gov/geo/) database serves as an external validation of the TCGA dataset and contains two subsets: GSE7390 and GSE42568. GSE42568 dataset contained 17 cases of paracancerous tissues and 104 cases of cancerous tissues. GSE7390 dataset only contained 198 cases of paracancerous tissues. Clinical information of the GEO test set is shown in Table 2. The RNA transcriptome data from different platforms were normalized using the “limma” and “sva” R packages.

**Table 2:** Clinical features of BRCA patients (*n* = 302) from GEO database

Variables	GSE7390		GSE42568	
	*N*	%	*N*	%
Total	198	100.0%	104	100.0%
Age, years				
<60	195	98.5%	59	56.7%
≥60	3	1.5%	45	43.3%
Grade				
G1	30	15.2%	11	10.5%
G2	83	41.9%	40	38.5%
G3	83	41.9%	53	51.0%
ER status				
Negative	64	32.3%	34	32.7%
Positive	134	67.7%	67	64.4%

### Expression of cancer driver genes in breast cancer tissues

The list of 568 cancer driver genes was obtained from the Integrative OncoGenomics platform (https://www.intogen.org/search). The differentially expressed genes (DEGs) were analyzed according to the classification of cancerous and paracancerous tissues by using the “limma” R package (*P*<0.05) in GSE42568 and TCG datasets. Venn diagrams were drawn to summarize differentially expressed genes both in TCGA and GEO datasets. These DEGs are considered to be associated with tumor progression and are used for further prognostic molecular screening.

### Construction and validation of the risk signature

We used the univariate Cox and Kaplan-Meier method to screen for cancer driver genes associated with overall survival (OS) (*P*<0.05) in breast cancer patients. Candidate genes that were both differentially expressed and associated with OS were substituted into least absolute shrinkage and selection operator (Lasso) Cox regression and stepwise multivariate Cox proportional regression to construct the risk signature. According to the risk driver genes expression of the prognostic signature, R package Stats was used for Principal Component Analysis (PCA). PCA could confirm the clustering ability of the cancer driver genes risk signature.

We used several methods to analyze the clinical value of the risk signature. Survival curves and receiver operating characteristic (ROC) curves were used to verify the prognostic value of different risk groups. Univariate and multifactorial regression analyses were performed to explore the independent prognostic role of risk scores. Besides, we further analyzed whether age, tumor stage, and other clinicopathology were associated with risk scores.

### Construction of transcription factor regulatory networks and functional enrichment analysis

To clarify the possible mechanisms by which cancer driver genes affect the progression and prognosis of breast cancer, we constructed a transcription factor regulatory network and gene set enrichment analysis (GSEA). The cancer-associated transcription factors were obtained from the Cistrome platform (http://cistrome.org/). We screened for cancer driver genes associated with transcription factors (|*R*^2^| > 0.3 and *P* < 0.05) and constructed a regulatory network using Cytoscape software (https://cytoscape.org/). Gene Ontology (GO) and Kyoto Encyclopedia of Genes and Genomes (KEGG) enrichment analyses were performed with the “enrichplot” R package, which suggests possible functional and pathway sets of cancer driver genes affecting breast cancer prognosis.

### Correlation analysis of tumor microenvironment

The immune and TME scores were calculated for each sample using single-sample GSEA (ssGSEA) algorithm by the “gsva” R package. We first analyzed the expression levels of PD-1, stromal score, immune score, and estimate score in different risk groups. We likewise analyzed the correlation of risk groups with 16 immune cell scores and 13 immune-related pathway scores. These results contribute to confirm the interaction of tumor microenvironment with cancer driver genes.

### Statistical analysis

All data processing was performed on R v3.4.1 (https://www.r-project.org/). Differences in gene expression between cancerous and paracancerous tissues were analyzed by the Wilcoxon method. The correlation between transcription factors and cancer driver gene expression was performed using the Pearson’s correlation coefficient method. Kaplan-Meier method verifies the impact of the candidate driver genes on patient OS. Mann–Whitney *U* test was used to compare immune or TME scores between the high-risk and low-risk groups. Univariate and multifactorial regression were used to analyze the prognostic value of risk scores.

## Results

### Differentially expressed cancer driver genes in TCGA and GEO datasets

**Fig. 1:**
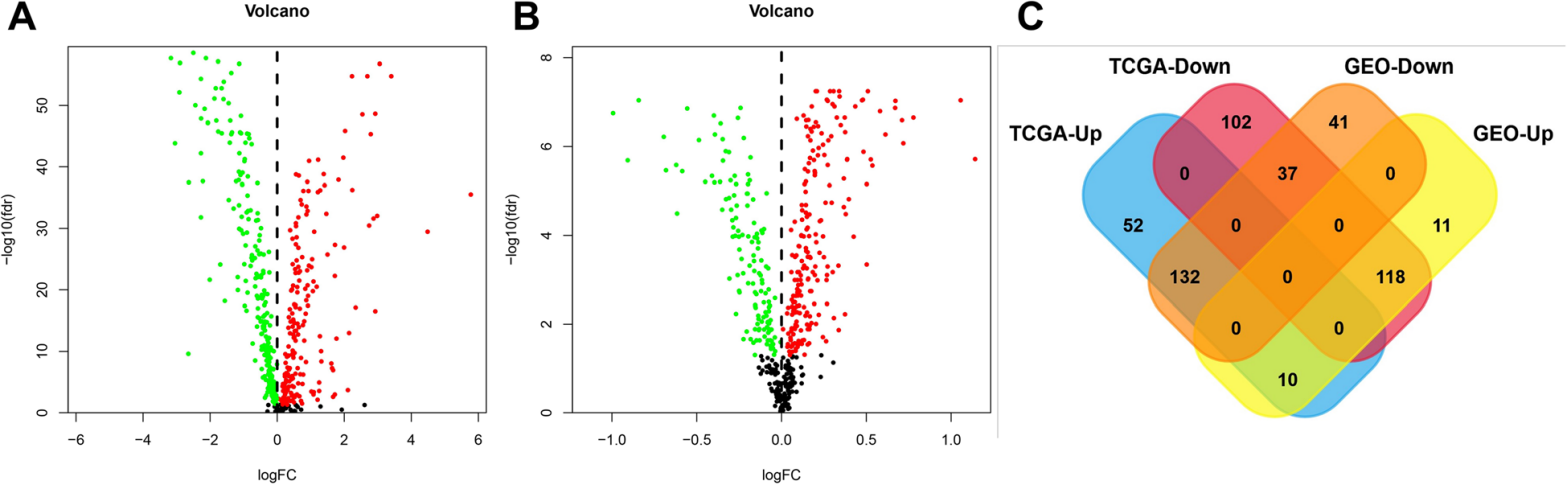
The differentially expressed genes in TCGA cohort and GEO cohort. **A** The expression of cancer driver genes between tumor tissues and paracancerous tissues in TCGA cohort. **B** The expression of cancer driver genes between tumor tissues and paracancerous tissues in GEO cohort. **C** Venn diagrams for differentially expressed genes

In the TCGA cohort, the expression of 194 cancer driver genes was higher in cancer tissues than in paracancerous tissues (*P*<0.05), and 257 cancer driver genes were lower in cancer tissues than in paracancerous tissues (*P*<0.05) (Fig. 1A). The results of the GEO cohort were consistent with the TCGA cohort, with 210 genes highly expressed and 139 genes lowly expressed in cancer tissues (*P*<0.05) (Fig. 1B, C). Most cancer driver genes are specifically expressed in cancerous tissues, which suggest that driver genes may be involved in multiple biological processes in breast cancer.

### Construction of a thirteen-mRNA signature for predicting patient prognosis

**Fig. 2:**
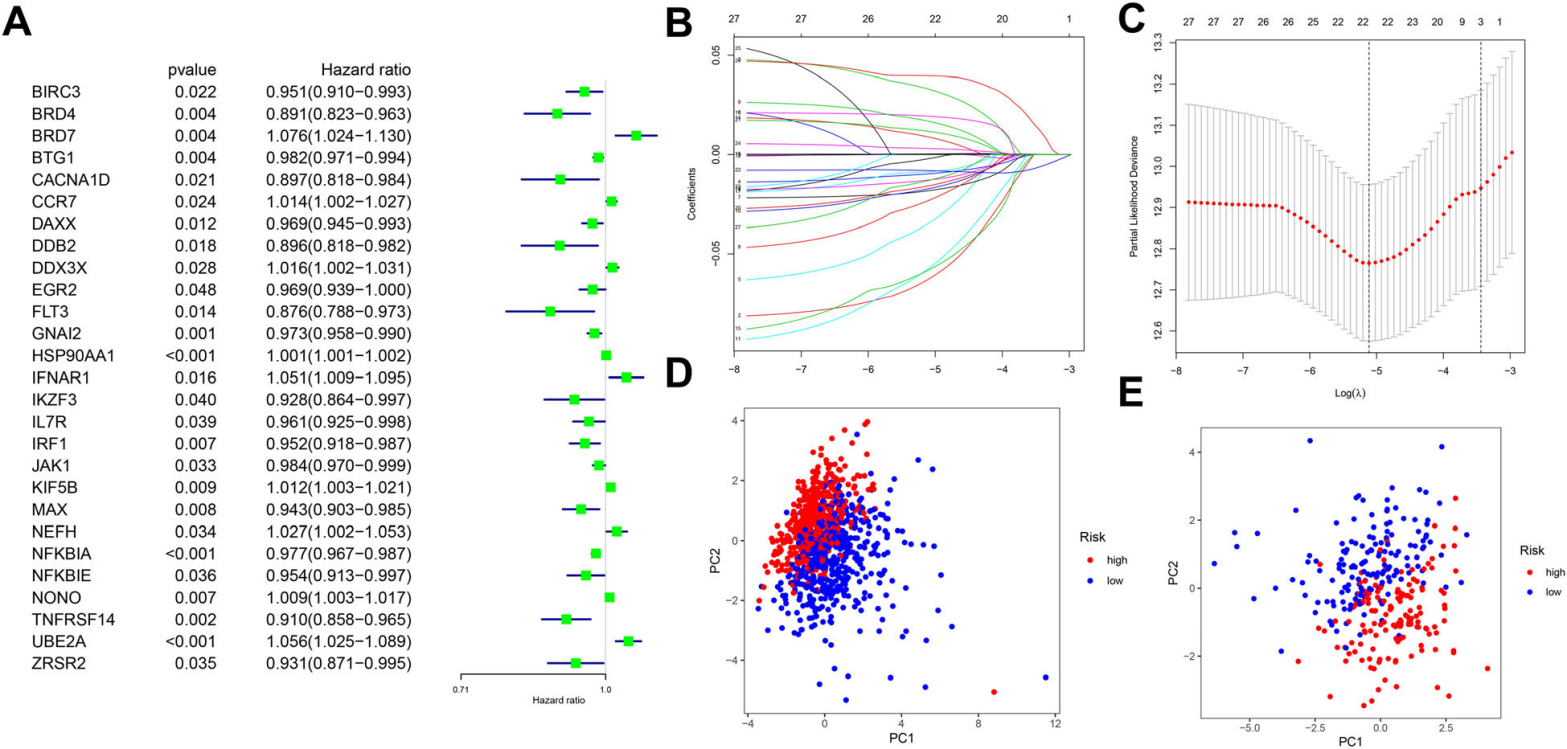
Construction and validation of the cancer driver gene signature. **A** Univariate regression screens prognosis-associated cancer driver genes. **B**, **C** Lasso regression screens candidate risk cancer driver genes. **D** PCA validation of the distribution of different risk groups in the training set. **E** PCA validation of the distribution of different risk groups in the test set

Through univariate Cox and Kaplan-Meier analysis (*P*<0.05), we screened 27 cancer driver genes associated with OS in breast cancer patients (Fig. 2A). Substituting these candidate molecules in lasso Cox regression and stepwise multivariate Cox proportional regression, we finally constructed a thirteen-mRNA risk signature (Fig. 2B, C). The coefficients for each risk genes are shown in Table 3. The risk score for each patient can be calculated by the risk formula, risk score= e ^(expression of BRD4*(−0.072) + expression of BRD7*0.049 + expression of BTG1*(−0.014) + expression of CCR7*0.023 + expression of DAXX*(−0.026) + expression of DDX3X*0.024 + expression of EGR2*(−0.024) + expression of FLT3*(−0.096) + expression of IKZF3*(−0.089) + expression of JAK1*(−0.020) + expression of MAX*(−0.034) + expression of NFKBIA*(−0.009) + expression of UBE2A*0.051)^. According to the median values of the risk scores in the TCGA cohort, all patients were divided into high-risk and low-risk groups. PCA validates that the different risk groups showed a two-way distribution, indicating the high specificity of the risk signature (Fig. 2D, E).

**Table 3:** Each gene of the thirteen-mRNA signature

mRNA	Coefficient	HR	95%CI	*P*
BRD4	−0.072	0.931	0.870–0.996	0.037
BRD7	0.049	1.050	0.989–1.116	0.109
BTG1	−0.014	0.986	0.974–0.998	0.019
CCR7	0.023	1.023	1.015–1.031	<0.001
DAXX	−0.026	0.975	0.950–0.999	0.049
DDX3X	0.024	1.024	1.007–1.041	0.007
EGR2	−0.024	0.976	0.948–1.006	0.115
FLT3	−0.096	0.909	0.824–1.002	0.054
IKZF3	−0.089	0.915	0.851–0.984	0.017
JAK1	−0.020	0.981	0.962–0.999	0.042
MAX	−0.034	0.967	0.925–1.010	0.132
NFKBIA	−0.009	0.991	0.980–1.002	0.092
UBE2A	0.051	1.052	1.012–1.094	0.010

**Fig. 3:**
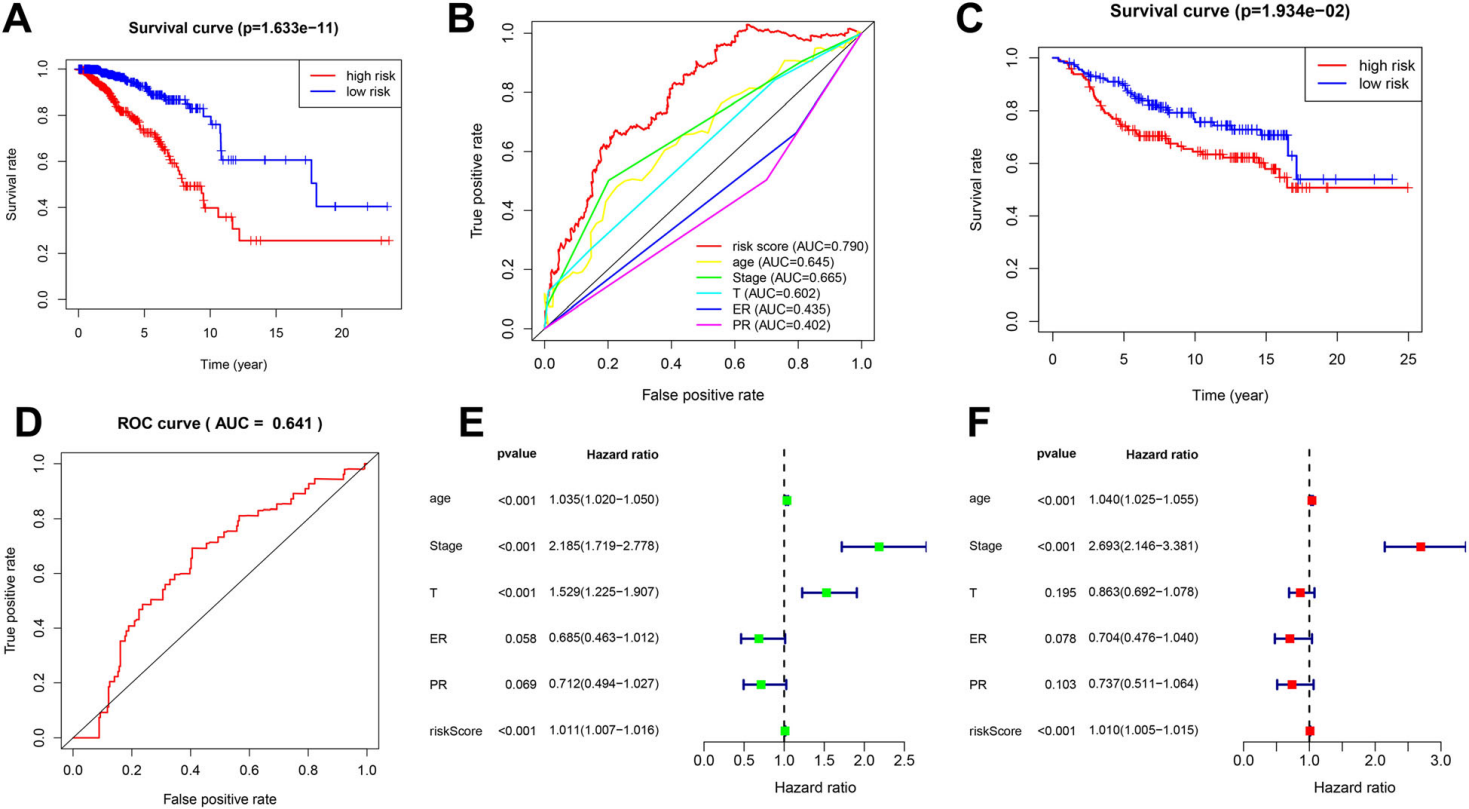
Survival analysis and prognostic value of the risk signature. **A** Survival analysis of different risk groups in the training set. **B** ROC curves validate the sensitivity and specificity of the risk signature in the training set. **C** Survival analysis of different risk groups in the test set. **D** ROC curves validate the sensitivity and specificity of the risk signature in the test set. **E** Univariate regression analysis of clinical characteristics and risk score in the TCGA cohort. **F** Multivariate regression analysis of clinical characteristics and risk score in the TCGA cohort

Survival curves were used to analyze the relationship between risk groups and OS of breast cancer patients. In the training set, the overall survival rate in the high-risk group was lower than in the low-risk group (*P*<0.05) (Fig. 3A). The area under curve (AUC) of the risk score (AUC 0.790) was higher than other clinical features, suggesting the high precision of the risk signature for predicting patient survival (Fig. 3B). The results of the test set were consistent with the training set. The survival of patients in different risk groups differed significantly (*P*<0.05), and the risk score could predict the survival of breast cancer patients well (AUC 0.641) (Fig. 3C, D). Univariate and multifactorial regression analyses suggested that risk score could be an independent risk factor affecting the prognosis of breast cancer patients (*P*<0.05) (Fig. 3E, F).

**Fig. 4:**
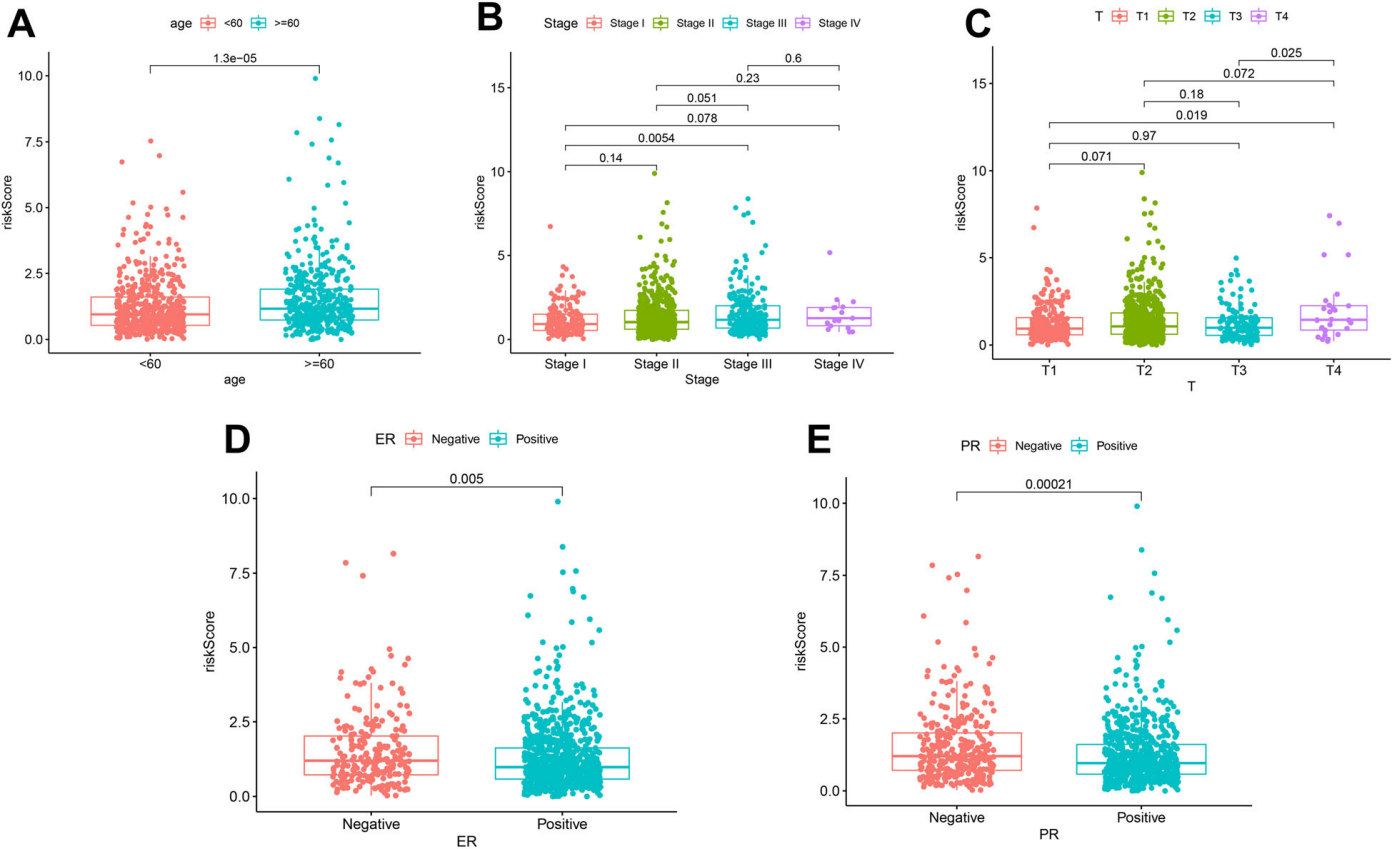
Correlation analysis of risk scores and clinical features. **A** Distribution of risk score in different age groups. **B** Distribution of risk score in different tumor stages. **C** Distribution of risk score in different T stage. **D** Distribution of risk score in different estrogen receptor status. **E** Distribution of risk score in different progesterone receptor status

We analyzed the level of risk scores in different clinical characteristics separately. The results suggest that people older than 60 years, advanced tumor stage, and estrogen and progesterone receptor-negative patients have higher risk scores (*P*<0.05), which may represent a poorer prognosis (Fig. 4). This group belongs to the high-risk group of the risk signature and requires more attention.

### Transcription factor regulatory networks and functional enrichment analysis

**Fig. 5:**
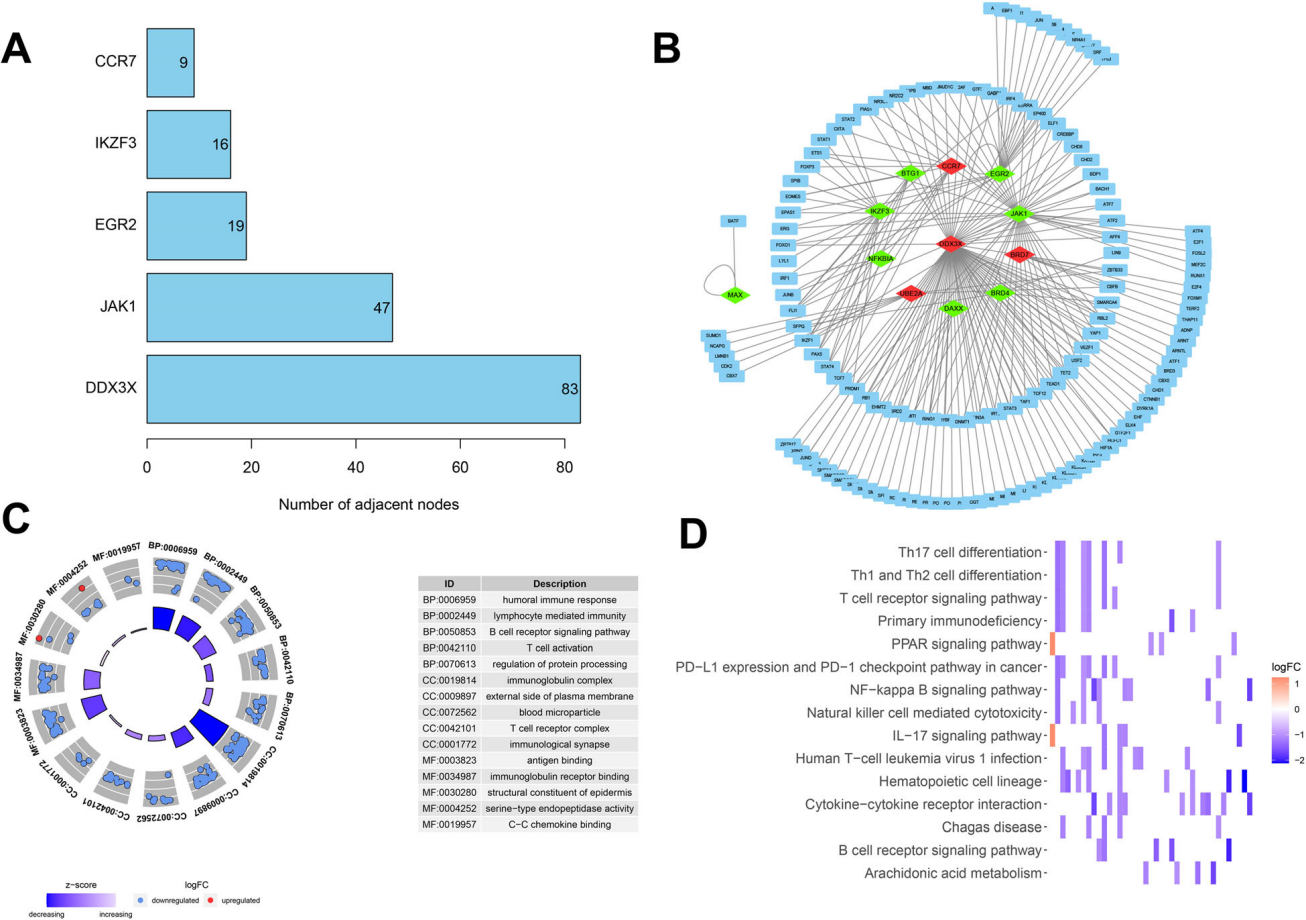
Transcription factor regulatory networks and functional enrichment analysis. **A** The number of cancer driver genes that are highly correlated with transcription factors. **B** Regulatory networks of transcription factors and cancer driver genes. **C** GO analysis of screened cancer driver genes. **D** KEGG analysis of screened cancer driver genes

To explore the possible mechanisms by which cancer driver genes affect breast cancer progression and prognosis, we first tried to explore the co-expression network of transcription factors and cancer driver genes. Through Pearson’s correlation coefficient analysis (|*R*^2^|> 0.3 and *P*< 0.05), 206 transcription factors were associated with cancer driver gene expression, and DDX3X, JAK1, EGR2, IKZF3, and CCR7 were the five most enriched cancer driver genes (Fig. 5A). A series of twelve cancer driver gene expressions were identified to be associated with transcription factors, of which CCR7, BRD7, DDX3X, and UBE2A were upregulated in breast cancer tissues, and the others were downregulated in breast cancer tissues. Next, all molecules were substituted into Cytoscape to map the regulatory network (Fig. 5B).

GO and KEGG analysis suggest possible mechanisms by which cancer driver genes impact breast cancer prognosis. GO analysis showed that the functional sets of immune response, T cell activity, and immune-related receptors were significantly enriched. KEGG analysis suggests that B cell receptor signaling pathway, T cell receptor signaling pathway, and PD−1 checkpoint pathway were significantly enriched. These results suggest a strong association between cancer driver genes and the immune status, which supports our further studies on the risk signature and tumor microenvironment.

### Correlation of the risk signature with tumor microenvironment

**Fig. 6:**
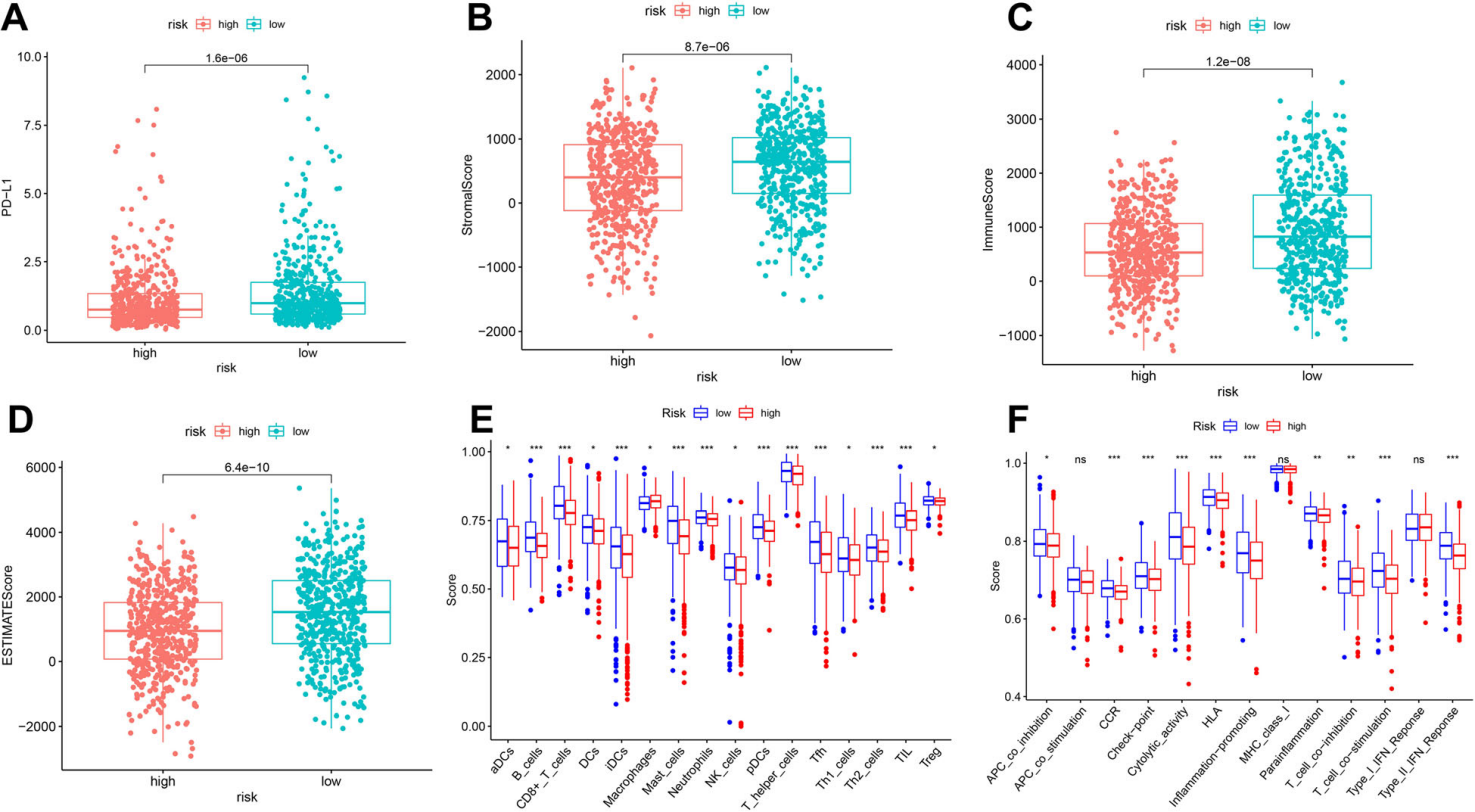
Correlation analysis of risk groups and tumor microenvironment. **A** Distribution of PD-L1 level in different risk groups. **B** Distribution of stromal score in different risk groups. **C** Distribution of immune score in different risk groups. **D** Distribution of estimate score in different risk groups. **E** Distribution of immune cell score in different risk groups. **F** Distribution of immune-related pathway score in different risk groups. *P<0.05, **P<0.01, and ***P<0.001

Alterations in the tumor microenvironment may be an essential way in which cancer driver genes affect breast cancer. We analyzed the correlation between PD-1 levels, tumor microenvironment, and risk groups. The results suggested that PD-1 expression levels, stromal scores, and immune scores were higher in the low-risk group compared to the high-risk group (*P*<0.05) (Fig. 6A-D). We likewise analyzed the correlation of immune cells and immune pathways with the risk signature, and the results suggested that most immune function scores differed significantly in the risk groups (*P*<0.05) (Fig. 6E, F).

## Discussion

In the precision medicine era, the mutation and expression characteristics of breast cancer patients’ tumor genomes are playing an increasingly critical role in the choice of therapeutic strategies [23]. Tumor heterogeneity is the main reason for the different responses to the treatment of breast cancer patients [24, 25]. Different molecular typing is used to explain breast cancer heterogeneity. For example, estrogen and progesterone receptor status can indicate whether a patient is suitable for endocrine therapy [26]. In contrast, traditional tumor markers are gradually showing their limitations, while novel markers at the molecular level, such as mRNA, miRNA, and DNA methylation, are being intensively investigated [27–30]. Tumor-infiltrating lymphocytes (TIL) are a heterogeneous population of lymphocytes [31]. Triple-negative breast cancers have the highest degree of TIL infiltration, followed by HER2+ breast cancers [32]. The KEYNOTE-086 study found that abundant mesenchymal TIL was closely associated with better efficacy of pembrolizumab [33]. The combined consideration of PD-L1 levels in tumor cells and immune cells predicts the possible benefit of receiving immunotherapy in breast cancer patients [34]. Molecular targeted therapy, immunotherapy, and novel molecular biomarkers have broadened the treatment opportunities for breast cancer, improving patients’ quality of life and prolonging survival time.

The specific expression of cancer driver genes is a major contributor to the formation of the tumor microenvironment, which further leads to various biological processes such as uncontrolled tumor cell proliferation, invasion, metastasis, recurrence, and drug resistance [21, 35]. Martínez’s study analyzed the gene expression of over 20,000 tumor samples and mapped cancer driver gene profiles [9]. Algorithms and identification of cancer driver genes have been intensively studied [36, 37]; however, the clinical value of newly identified cancer driver genes has been relatively under-explored. Aberrant expression and mutations of cancer driver genes are very common in breast cancer patients, approaching 50% [12]. Comprehensive analysis of the clinical significance of these genes may lead to the discovery of new clinical biomarkers. We studied cancer driver gene expression profiles of more than 1000 breast cancer samples and identified cancer driver genes associated with breast cancer progression and prognosis. For example, Rizeq’s study found that activation of the C–C chemokine receptor 7 (CCR7)-related complex increased tumor cell proliferation and migration [38]. Vahidi’s study concluded that CD8-positive memory T cells in tumor-draining lymph nodes are associated with CCR7 [39]. We found the specific expression and prognostic value of CCR7 in breast cancer. Both Niu [40] and our study confirmed the oncogenic role of bromodomain-containing 7 (BRD7) molecule in breast cancer. In general, these studies suggest the involvement of cancer driver genes in complex tumor biological behavior and clinically important roles. Based on the identification of the cancer driver genes, we further constructed a thirteen-mRNA prognostic signature with high specificity and sensitivity of prediction. The survival of patients in different risk groups differed significantly, and the risk score was an independent risk factor in predicting breast cancer prognosis.

Several clinical trials have demonstrated favorable results of PD-1/PD-L1 antibodies in the treatment of breast cancer [34, 41, 42]. Targeting PD-1-related pathways effectively blocks immune surveillance and escape and activates the immune response of T cells against tumor cells [43, 44]. The stromal environment, immune cell level, and composition are crucial in the immunotherapy process. Cancer driver genes can indirectly alter the tumor microenvironment through multiple regulatory networks [21], but fewer studies have been conducted. Raskov’s study [45] found that mutations in some cancer driver genes could increase accessibility for DNA targeting chemotherapeutics and reduce cytotoxic drug resistance in colorectal cancer. In our study, KEGG analysis revealed significant enrichment of immune-related pathways such as T cell activation and PD-1-related pathways, suggesting possible interactions between cancer driver genes and immunity. We found that the survival time was longer in the low-risk group compared to the high-risk group, who also had higher PD-1 expression, immune-related scores, and tumor microenvironment scores. Our findings suggest that cancer driver genes may be involved in the body’s immune response and have an enhancing effect on the effectiveness of immunotherapy.

## Conclusion

In summary, we analyzed the expression profiles of 568 cancer driver genes in breast cancer and identified driver genes associated with breast cancer prognosis. We further constructed a risk signature to predict breast cancer prognosis, and both the training and external test sets performed sensitive predictive efficacy. Cancer driver genes may affect the biological behavior of breast cancer by mediating transcriptional processes and alterations in the immune and tumor microenvironment. Our study confirms the critical role of cancer driver genes in breast cancer, and it is expected to supply a reference for the prognostic stratification and treatment strategy of breast cancer.

## Data Availability

All data was obtained from The Cancer Genome Atlas (https://portal.gdc.cancer.gov/) and Gene Expression Omnibus (https://www.ncbi.nlm.nih.gov/geo/) databases.

## References

[1] Siegel RL, Miller KD, Fuchs HE, Jemal A (2021). Cancer statistics, 2021. CA Cancer J Clin..

[2] Guo Y, Mao X, Qiao Z, Chen B, Jin F (2020). A novel promoter CpG-based signature for long-term survival prediction of breast cancer patients. Front Oncol..

[3] Liang F, Qu H, Lin Q, Yang Y, Ruan X, Zhang B, Liu Y, Yu C, Zhang H, Fang X, Hao X (2015). Molecular biomarkers screened by next-generation RNA sequencing for non-sentinel lymph node status prediction in breast cancer patients with metastatic sentinel lymph nodes. World J Surg Oncol..

[4] Prejac J, Dedić Plavetić N, Gotovac Jerčić K, Borovečki F (2021). A first report of a rare TP53 variant associated with Li-Fraumeni syndrome manifesting as invasive breast cancer and malignant solitary fibrous tumor. World J Surg Oncol..

[5] Dieci MV, Miglietta F, Guarneri V (2021). Immune infiltrates in breast cancer: recent updates and clinical implications. Cells.

[6] Jia R, Li Z, Liang W, Ji Y, Weng Y, Liang Y, Ning P (2020). Identification of key genes unique to the luminal a and basal-like breast cancer subtypes via bioinformatic analysis. World J Surg Oncol..

[7] Yuan Q, Zheng L, Liao Y, Wu G (2021). Overexpression of CCNE1 confers a poorer prognosis in triple-negative breast cancer identified by bioinformatic analysis. World J Surg Oncol..

[8] Liu C, Li Y, Wei M, Zhao L, Yu Y, Li G (2019). Identification of a novel glycolysis-related gene signature that can predict the survival of patients with lung adenocarcinoma. Cell Cycle..

[9] Martínez-Jiménez F, Muiños F, Sentís I, Deu-Pons J, Reyes-Salazar I, Arnedo-Pac C, Mularoni L, Pich O, Bonet J, Kranas H, Gonzalez-Perez A, Lopez-Bigas N (2020). A compendium of mutational cancer driver genes. Nat Rev Cancer..

[10] Bossi D, Cicalese A, Dellino GI, Luzi L, Riva L, D'Alesio C, Diaferia GR, Carugo A, Cavallaro E, Piccioni R, Barberis M, Mazzarol G, Testori A, Punzi S, Pallavicini I, Tosti G, Giacó L, Melloni G, Heffernan TP, Natoli G, Draetta GF, Minucci S, Pelicci PG, Lanfrancone L (2016). In vivo genetic screens of patient-derived tumors revealed unexpected frailty of the transformed phenotype. Cancer Discov..

[11] Zhao S, Liu J, Nanga P, Liu Y, Cicek AE, Knoblauch N, He C, Stephens M, He X (2019). Detailed modeling of positive selection improves detection of cancer driver genes. Nat Commun..

[12] Zhang G, Wang Y, Chen B, Guo L, Cao L, Ren C, Wen L, Li K, Jia M, Li C, Mok H, Chen X, Wei G, Lin J, Zhang Z, Hou T, Han-Zhang H, Liu C, Liu H, Liu J, Balch CM, Meric-Bernstam F, Liao N (2019). Characterization of frequently mutated cancer genes in Chinese breast tumors: a comparison of Chinese and TCGA cohorts. Ann Transl Med..

[13] Krøigård AB, Larsen MJ, Lænkholm AV, Knoop AS, Jensen JD, Bak M, Mollenhauer J, Thomassen M, Kruse TA (2018). Identification of metastasis driver genes by massive parallel sequencing of successive steps of breast cancer progression. PLoS One..

[14] Cui Y, Guo G (2016). Immunomodulatory function of the tumor suppressor p53 in host immune response and the tumor microenvironment. Int J Mol Sci.

[15] Yaghoobi H, Azizi H, Oskooei VK, Taheri M, Ghafouri-Fard S (2018). Assessment of expression of interferon γ (IFN-G) gene and its antisense (IFNG-AS1) in breast cancer. World J Surg Oncol..

[16] Kim J (2021). In silico analysis of differentially expressed genesets in metastatic breast cancer identifies potential prognostic biomarkers. World J Surg Oncol..

[17] Zhou X, Xiao C, Han T, Qiu S, Wang M, Chu J, Sun W, Li L, Lin L (2020). Prognostic biomarkers related to breast cancer recurrence identified based on Logit model analysis. World J Surg Oncol..

[18] Bianchi JJ, Zhao X, Mays JC, Davoli T (2020). Not all cancers are created equal: tissue specificity in cancer genes and pathways. Curr Opin Cell Biol..

[19] Liu WJ, Du Y, Wen R, Yang M, Xu J (2020). Drug resistance to targeted therapeutic strategies in non-small cell lung cancer. Pharmacol Ther..

[20] Hwang KT, Kim BH, Oh S, Park SY, Jung J, Kim J, Choi IS, Jeon SY, Kim WY (2019). Prognostic role of KRAS mRNA expression in breast cancer. J Breast Cancer..

[21] Wang G, Wang Q, Liang N, Xue H, Yang T, Chen X, Qiu Z, Zeng C, Sun T, Yuan W, Liu C, Chen Z, He X (2020). Oncogenic driver genes and tumor microenvironment determine the type of liver cancer. Cell Death Dis..

[22] Li G, Du X, Wu X (2021). Large-scale transcriptome analysis identified a novel cancer driver genes signature for predicting the prognostic of patients with hepatocellular carcinoma. Front Pharmacol..

[23] Zhu C, Hu H, Li J, Wang J, Wang K, Sun J (2020). Identification of key differentially expressed genes and gene mutations in breast ductal carcinoma in situ using RNA-seq analysis. World J Surg Oncol..

[24] Hanna WM, Rüschoff J, Bilous M, Coudry RA, Dowsett M, Osamura RY, Penault-Llorca F, van de Vijver M, Viale G (2014). HER2 in situ hybridization in breast cancer: clinical implications of polysomy 17 and genetic heterogeneity. Mod Pathol..

[25] Liu X, Jin G, Qian J, Yang H, Tang H, Meng X, Li Y (2018). Digital gene expression profiling analysis and its application in the identification of genes associated with improved response to neoadjuvant chemotherapy in breast cancer. World J Surg Oncol..

[26] Bianchini G, Balko JM, Mayer IA, Sanders ME, Gianni L (2016). Triple-negative breast cancer: challenges and opportunities of a heterogeneous disease. Nat Rev Clin Oncol..

[27] Mao XH, Ye Q, Zhang GB, Jiang JY, Zhao HY, Shao YF, Ye ZQ, Xuan ZX, Huang P (2021). Identification of differentially methylated genes as diagnostic and prognostic biomarkers of breast cancer. World J Surg Oncol..

[28] Ghafouri-Fard S, Oskooei VK, Azari I, Taheri M (2018). Suppressor of cytokine signaling (SOCS) genes are downregulated in breast cancer. World J Surg Oncol..

[29] Mohamadalizadeh-Hanjani Z, Shahbazi S, Geranpayeh L (2020). Investigation of the SPAG5 gene expression and amplification related to the NuMA mRNA levels in breast ductal carcinoma. World J Surg Oncol..

[30] Yu YZ, Mu Q, Ren Q, Xie LJ, Wang QT, Wang CP (2021). miR-381-3p suppresses breast cancer progression by inhibition of epithelial-mesenchymal transition. World J Surg Oncol.

[31] Criscitiello C, Bagnardi V, Pruneri G, Vingiani A, Esposito A, Rotmensz N, Curigliano G (2017). Prognostic value of tumour-infiltrating lymphocytes in small HER2-positive breast cancer. Eur J Cancer..

[32] Stanton SE, Disis ML (2016). Clinical significance of tumor-infiltrating lymphocytes in breast cancer. J Immunother Cancer..

[33] Adams S, Loi S, Toppmeyer D, Cescon DW, de Laurentiis M, Nanda R, Winer EP, Mukai H, Tamura K, Armstrong A, Liu MC, Iwata H, Ryvo L, Wimberger P, Rugo HS, Tan AR, Jia L, Ding Y, Karantza V, Schmid P (2019). Pembrolizumab monotherapy for previously untreated, PD-L1-positive, metastatic triple-negative breast cancer: cohort B of the phase II KEYNOTE-086 study. Ann Oncol..

[34] Schmid P, Rugo HS, Adams S, Schneeweiss A, Barrios CH, Iwata H, Diéras V, Henschel V, Molinero L, Chui SY, Maiya V, Husain A, Winer EP, Loi S, Emens LA, IMpassion130 Investigators (2020). Atezolizumab plus nab-paclitaxel as first-line treatment for unresectable, locally advanced or metastatic triple-negative breast cancer (IMpassion130): updated efficacy results from a randomised, double-blind, placebo-controlled, phase 3 trial. Lancet Oncol..

[35] Matter MS, Marquardt JU, Andersen JB, Quintavalle C, Korokhov N, Stauffer JK, Kaji K, Decaens T, Quagliata L, Elloumi F, Hoang T, Molinolo A, Conner EA, Weber A, Heikenwalder M, Factor VM, Thorgeirsson SS (2016). Oncogenic driver genes and the inflammatory microenvironment dictate liver tumor phenotype. Hepatology..

[36] Guo WF, Zhang SW, Zeng T, Li Y, Gao J, Chen L (2019). A novel network control model for identifying personalized driver genes in cancer. PLoS Comput Biol..

[37] Guo WF, Zhang SW, Zeng T, Akutsu T, Chen L (2020). Network control principles for identifying personalized driver genes in cancer. Brief Bioinform..

[38] Rizeq B, Malki MI (2020). The role of CCL21/CCR7 chemokine axis in breast cancer progression. Cancers (Basel).

[39] Vahidi Y, Bagheri M, Ghaderi A, Faghih Z (2020). CD8-positive memory T cells in tumor-draining lymph nodes of patients with breast cancer. BMC Cancer..

[40] Niu W, Luo Y, Zhou Y, Li M, Wu C, Duan Y, Wang H, Fan S, Li Z, Xiong W, Li X, Li G, Ren C, Li H, Zhou M (2020). BRD7 suppresses invasion and metastasis in breast cancer by negatively regulating YB1-induced epithelial-mesenchymal transition. J Exp Clin Cancer Res..

[41] Schmid P, Cortes J, Pusztai L, McArthur H, Kümmel S, Bergh J, Denkert C, Park YH, Hui R, Harbeck N, Takahashi M, Foukakis T, Fasching PA, Cardoso F, Untch M, Jia L, Karantza V, Zhao J, Aktan G, Dent R, O’Shaughnessy J (2020). Pembrolizumab for early triple-negative breast cancer. N Engl J Med..

[42] Li X, Lian Z, Wang S, Xing L, Yu J (2018). Interactions between EGFR and PD-1/PD-L1 pathway: implications for treatment of NSCLC. Cancer Lett..

[43] Cabo M, Offringa R, Zitvogel L, Kroemer G, Muntasell A, Galluzzi L (2017). Trial Watch: Immunostimulatory monoclonal antibodies for oncological indications. Oncoimmunology..

[44] Topalian SL, Drake CG, Pardoll DM (2015). Immune checkpoint blockade: a common denominator approach to cancer therapy. Cancer Cell..

[45] Raskov H, Søby JH, Troelsen J, Bojesen RD, Gögenur I (2020). Driver gene mutations and epigenetics in colorectal cancer. Ann Surg..

